# Mortality of Surgical Operations in the Upper Lake States, Compared with That of Other Regions

**Published:** 1876-09

**Authors:** Edmund Andrews

**Affiliations:** Professor of Princples and Practice of Surgery in Chicago Medical College


### THE MORTALITY OF SURGICAL OPERATIONS IN THE UPPER LAKE STATES, COMPARED WITH THAT OF OTHER REGIONS.

By EDMUND ANDREWS, A.M., M.D.,

Professor of Principles and Practice of Surgery in Chicago Medical College,

Assisted by Thos. B. Lacey, M.D.,

Assistant Surgeon in the National Soldiers' Home.

(Continued.)

### AMPUTATION OF THE THIGH.

This important operation furnishes us the following list of cases in the Lake States:

### TABLE VIII.

### AMPUTATION OF THE THIGH.

| No. Andrew's Sur. Rec | OPERATOR. | Age. | REASON FOR OPERATION. | COMPLICATIONS. | Operati'n | Cond. at opr | Time to operation. | Result. | Time to death or recovery | Practice. |
|---|---|---|---|---|---|---|---|---|---|---|
| 5471 | Dr. E. Andrews | 16 | Protrusion, of bone after prev. amput | | Re-a. m. 3 | Good | Secondary | Recover'd | | Hospital. |
| | " E. W. Lee | 22 | Comp. fract. of leg | | Flap low3 | Med. | " | Died | 9 days | Private |
| | " " " | 10 | " " " | | " " | Good | Primary | Recover'd | 3 weeks. | " |
| 8880 | " E. Andrews | 9 | Leg crushed by cars | Great shock | Lower 3d | Bad. | " | Died | | Hospital. |
| 7726 | " " " | 50 | Carious knee | Much exhausted | Flap. m. 3 | " | 2½ years | " | 6 days | Private |
| 7727 | " " " | 20 | " " | None | " " | Good | 6 " | Recover'd | | " |
| 7752 | Not stated | 30 | " " | Suspected phthisis | Lower 3d | Med. | 8 " | " | | " |
| | " S. S. Bedal | 64 | Comp. fract. thigh | Gangrene | Mid. 3 cir. | Good | 10 days | " | | " |
| | " Thos. Lacy | .. | Disease of knee | | " " | | 13 years | " | | " |
| 775 | " E. Andrews | 17 | Gangrene from injury | None | Flap. low3 | Good | 6 days | " | | Private |
| 1612 | " " " | 40 | Comp. fract. of knee | Intemperate | " " | " | Primary | " | | " |
| 1653 | " " " | .. | Knee crushed by timber | None | " " | " | " | " | | " |
| 5488 | " " " | .. | Caries of knee | Had been previous exsec. | " " | | | " | 6 mos. | Hospital. |
| | " J. S. Sherman | .. | " " | | " " | Bad. | | " | 5 weeks | " |
| 1369 | " E. Andrews | 36 | R. R. accident, drunk | Crushed thigh and op. leg | Am. " | " | Primary | Died | 1 hour | Private |
| 6397 | " " " | 5 | Comp. fract. leg | Opposite knee bruised | Flap. low3 | Med. | Secondary | Recover'd | 10 weeks | Hospital. |
| | " M. Gunn | 50 | Senile gangrene | Intemperate | " " | " | 6 days | " | | Private |
| | " R. Isham | .. | Comp. fract. of femur | | " " | " | Primary | " | 7 weeks | Hospital. |
| | " La Count | 50 | " " knee | None | " " | Bad. | 36 hours | Died | 48 hours | Private |
| | " H. A. Johnson | 40 | Chronic inflam. of knee | " | " " | Good | Some mos. | Recover'd | | " |
| | " E. Andrews | .. | Crushed knee and hemorrhage | Great shock | " " | Bad. | Primary | Died | 1 hour | Hospital. |
| | " J. W. Freer | 33 | Cancer of knee | | Lower 3d | | | Recover'd | | " |
| | " G. Ammerman | 25 | Comp. and commin. fract. of leg | | " " | | Primary | " | 10 weeks | " |
| | " " " | 7 | " " " " " | Wounds op. limb, g. shock | " " | Bad. | " | Died | | " |
| | " A. Fisher | 10 | Cancer of tibia | | " " | " | 1 year | Recover'd | | Private |
| | " " " | 19 | Caries of knee and morti. of leg | | " " | " | 9 months | " | 7 weeks. | " |
| | " " " | 25 | " " " | | " " | " | 2 years | " | 6 " | " |
| | " " " | 8 | Freezing of leg | | " " | Med. | 6 months | " | 5 " | " |
| | " " " | 39 | Chronic inflam. of knee with caries | | " " | " | 2½ years | " | 7 " | " |
| | " " " | 30 | " " " | | " " | Bad. | 3 " | " | 10 " | " |
| | " " " | 20 | Knee joint nearly severed with ax. | | " " | " | 6 hours | " | 6 " | " |
| | " " " | 40 | Gunshot wound of knee joint | Inflamed—suppurating | " " | " | 3 weeks | " | 8 " | " |
| | " Hyde | 41 | Comp. and commin. fract. leg | None | " " | Good | Primary | " | 2 mos. | " |
| | " J. H. Hollister | 7 | Knee crushed by car wheel | " | " " | " | " | " | | " |
| | " E. Owens | .. | | | " " | | | " | | Hospital. |

### TABLE VIII.—Continued.

| | | | | | | | | | | |
|---|---|---|---|---|---|---|---|---|---|---|
| -------- | " " " ---- | .. | Disease of parts | -------- | " ." | ------ | -------- | " | -------- | " |
| -------- | " H. Wardner | 14 | Necrosis of entire tibia | Scrofulous | " " | Bad | 2 years | " | 2 mos. | Private |
| -------- | " M. Waterhouse | 25 | Diseased knee (chronic) | Great exhaustion | " " | " | -------- | " | -------- | " |
| -------- | " " " | 46 | Chronic suppuration of knee | Anæmia | " " | " | 3 years | " | 15 days | " |
| -------- | " E. D. Kittoe | 40 | Both knees and legs crushed | Hæmorrhage and shock | Both l. 3d | " | 2 hours | Died | 4 hours | " |
| -------- | " " " | 83 | Crushed ankle and split tibia | Great shock | Lower 3d | Med. | 1 hour | Recover'd | -------- | " |
| -------- | " " " | 14 | Necrosis of tibia and fibula | Debilitated | " " | Bad | 3 years | " | -------- | " |
| -------- | " " " | 24 | Crushed knee and part of thigh | Great shock | " " | " | Primary | Died | 16 hours | " |
| -------- | " J. Andrews | 22 | Scrofulous knee | -------- | " " | med. | 2 years | Recover'd | -------- | " |
| -------- | " " " | 34 | Fract. leg and thigh | Other injuries—heart dis. | -------- | Bad | Secondary | Died | 3 days | " |
| 5332 | " E. Andrews | 28 | Caries of knee after fracture | None | Flap. m. 3 | " | " | " | 2 mos. | Hospital. |
| -------- | " J. H. Hollister | 22 | Caries femur after amp. lower 3d. | Debility | Re-a. m. 3 | " | " | Recover'd | -------- | Private |
| -------- | " " | 22 | Above case relapsed 4 months after | " | " " | " | " | " | -------- | " |
| 7822 | " E. Andrews | 25 | Round-celled sarcoma of femur | Femur fract. in walking | Cir. up. 3 | Good | 6 years | " | -------- | " |
| 8748 | " " " | 34 | Caries of knee | Abscess in the bone | Flap. l. 3d | Med. | Many mos. | " | -------- | Hospital. |
| -------- | " S. Marks | 34 | Colloid—head tibia and femur | Not stated | Cir. low. 3 | Good | 6 months | " | 6 weeks | " |
| -------- | " " " | 10 | Dog bite followed by gangrene | " . " | Flap. " | Bad | 3 " | " | 3 " | " |
| -------- | " " " | 38 | Tetanus from injury of the thigh | " " | Flap. up 3 | " | 10 days | Died | 3 days | " |
| -------- | " " " | 24 | Traumatic Gangrene | " " | " low. 3 | " | 8 " | Recover'd | -------- | Private |
| -------- | " " " | 17 | Anchylosis knee, disease of tibia | " " | " " | Good | 9 years | " | -------- | " |
| -------- | " " " | 28 | Knee crushed by R. R. accident | " " | " up. 3 | Bad | 4 hours | " | 2 mos. | " |
| -------- | " " " | 9 | Leg crushed between cars | " " | " low. 3 | " | 8 hours | " | 6 weeks | " |
| -------- | " " " | 58 | Osteo-sarcoma of tibia | " " | " mid. 3 | Med. | 6 months | " | 4 " | " |
| -------- | " " " | 24 | Leg and lower thigh crushed | " " | " up. 3 | Bad | 12 hours | " | 8 mos. | Hospital. |
| -------- | " " " | 45 | Leg crushed by R. R. accident | " " | " low. 3 | Good | Primary | " | 6 weeks | Private |
| -------- | " " " | 19 | Anchylosis knee—disea. bones | " " | " mid. 3 | Bad | 6 years | " | 4 mos. | " |
| -------- | " " " | 32 | Disease of tibia and femur | " " | " low. 3 | Med. | 3 " | " | 5 " | Hospital. |
| -------- | " " " | 29 | Anchyl. knee, caries tibia | " " | " mid. 3 | " | 21 " | " | 2 " | Private |
| -------- | " " " | 30 | Knee joint opened with ax | " " | " " | Bad | 20 days | " | 6 " | " |
| -------- | " " " | 40 | Leg and knee crushed by rock | " " | " low. 3 | Good | Primary | " | 4 weeks | " |
| -------- | " " " | 16 | Leg crushed with R. R. accident | " " | " " | " | " | " | -------- | " |
| -------- | " " " | 59 | Osteo-sarcoma, tibia | " " | Middle 3d | Bad | About 1 yr. | Died | 2 weeks | " |
| -------- | " J. H. Hollister | 22 | Knee crushed by car wheel | Great shock | " " | " | Primary | " | -------- | " |
| -------- | " " " | 19 | " " " " " | " " | " " | Good | " | Recover'd | -------- | " |
| -------- | Cook Co. Hosp. | .. | -------- | -------- | " " | ------ | " | Died | -------- | Hospital. |
| -------- | Dr. E. D. Kittoe | 28 | Knee and part thigh crushed | Great shock | " " | Bad | " | " | 10 hours | Private |
| -------- | " A. Fisher | 10 | Comp. and commin. fract. of femur | -------- | Upper " | Good | 24 hours | Recover'd | 5 weeks | " |
| -------- | " " " | 26 | " " " " | Great shock | " " | Bad | 6 " | Died | 6 hours | " |
| -------- | " J. H. Hollister | 22 | Caries of femur | -------- | " " | " | -------- | Recover'd | -------- | " |
| -------- | " H. Wardner | 29 | Thigh crushed on R. R. | Great shock | " " | " | Primary | Died | 12 hours | " |

RECAPITULATION.

| | CASES. | DIED. | PER CENT. MORTALITY. |
|---|---|---|---|
| Total of all kinds | 76 | 18 | 24 |
| Traumatic, primary, upper 3d | 5 | 2 | 40 |
| " intermediary or secondary upper 3d | 1 | 1 | |
| Pathological, upper 3d | 2 | 0 | |
| Traumatic, primary, middle 3d | 4 | 3 | |
| " intermediary and secondary combined, middle 3d | 5 | 1 | |
| Pathological, middle 3d | 7 | 2 | |
| Traumatic, primary, lower 3d | 18 | 5 | 28 |
| " intermediary and secondary combined, lower 3d | 9 | 3 | 33 |
| Pathological, lower 3d | 22 | 0 | 0 |
| Hospital cases | 20 | 6 | 30 |
| Private practice | 54 | 11 | 20 |

There is on record in the literature of surgery a prodigious mass of cases of amputation of the thigh, but, unfortunately, most of them are so destitute of details that they cannot be properly classified. Generally there is no statement in what portion of the thigh the operation took place, and often the essential distinction into primary, secondary and pathological cases is ignored. The statistics of the upper two-thirds are especially meagre.

AMPUTATION UPPER 3D OF THIGH ABROAD, TRAUMATIC PRIMARY.

| AUTHORITIES. | CASES. | DIED. |
|---|---|---|
| Rept. Boston City Hosp., Dr. Cheever | 11 | 11 |
| Mass. Gen. Hosp. Rept., 1871 | 13 | 7 |
| Dr. Herrgolt, Siege of Strassburg, 1870–71 | 2 | 1 |
| Dr. Nunneley, Leeds Gen. Infirmary | 9 | 2 |
| Dr. E. Warren's Surg., p. 395, Confed. Army | 5 | 3 |
| Totals | 40 | 24 |

Mortality abroad, 60 per cent.

AMPUTATION UPPER 3D OF THIGH ABROAD, INTERMEDIARY AND SECONDARY COMBINED.

| AUTHORITIES. | CASES. | DIED. |
|---|---|---|
| Circular No. 3, Surg. Gen. U. S. A. | 7 | 2 |
| Dr. Herrgolt, Siege of Strassburg, 1870–71 | 6 | 5 |
| Dr. E. Warren's Surg., p. 395, Confederate Army | 5 | 1 |
| Totals | 18 | 8 |

Mortality abroad, 44 per cent.

AMPUTATION UPPER 3D OF THIGH ABROAD, PATHOLOGICAL.

| AUTHORITIES. | CASES. | DIED. |
|---|---|---|
| Circular No. 3, Surg. Gen. U. S. A. | 3 | 1 |
| Rept. Bost. City Hosp., Dr. Cheever | 2 | 1 |
| " Mass. Gen. Hosp., 1871 | 15 | 4 |
| " U. S. Marine Hosps., Dr. Woodworth | 1 | 0 |
| Leeds Gen. Infirmary, Mr. Nunneley | 9 | 2 |
| Totals | 30 | 8 |

Mortality abroad, 27 per cent.

AMPUTATION MIDDLE 3D OF THIGH ABROAD, TRAUMATIC PRIMARY.

| AUTHORITIES. | CASES. | DIED. |
|---|---|---|
| Circular No. 3, Surg. Gen. U. S. A. | 2 | 1 |
| Rept. Boston City Hosp., Dr. Cheever | 4 | 4 |
| " Mass. Gen. Hosp., 1871 | 13 | 6 |
| Dr. Herrgolt, Siege of Strassburg, 1870–71 | 1 | 1 |
| Dr. E. Warren's Surg., p. 395, Confederate Army U. S. | 13 | 4 |
| Totals | 33 | 16 |

Mortality abroad, 48 per cent.

AMPUTATION MIDDLE 3D OF THIGH ABROAD, TRAUMATIC, INTERMEDIARY AND SECONDARY COMBINED.

| AUTHORITIES. | CASES. | DIED. |
|---|---|---|
| Circular No. 3, Surg. Gen. U. S. A. | 4 | 1 |
| Rept. Boston City Hosp., Dr. Cheever | 4 | 1 |
| " Mass. Gen. Hosp, 1871 | 7 | 3 |
| Dr. Herrgolt, Siege of Strassburg, 1870–71 | 9 | 8 |
| Dr. E. Warren's Surg., p. 395, Confederate Army U. S. | 15 | 10 |
| Totals | 39 | 23 |

Mortality abroad, 59 per cent.

AMPUTATION MIDDLE 3D OF THIGH ABROAD, PATHOLOGICAL.

| AUTHORITIES. | CASES. | DIED. |
| --- | --- | --- |
| Circular No. 3, Surg. Gen. U. S. A. | 4 | 1 |
| Rept. Boston City Hosp., Dr. Cheever | 4 | 1 |
| " Mass. Gen. " 1871 | 47 | 10 |
| " Rostoch " | 1 | 0 |
| Totals | 56 | 12 |

Mortality abroad, 22 per cent,

AMPUTATION LOWER 3D OF THIGH ABROAD, TRAUMATIC, PRIMARY.

| AUTHORITIES. | CASES. | DIED. |
| --- | --- | --- |
| Circular No. 3, Surg. Gen. U. S. A. | 6 | 3 |
| Rept. Boston City Hosp., Dr. Cheever | 13 | 10 |
| " Mass. Gen. Hosp., 1871 | 35 | 12 |
| Dr. Herrgolt, Siege of Strassburg, 1870–71 | 2 | 2 |
| Dr. E. Warren's Surg. Confederate Army | 27 | 10 |
| Totals | 83 | 37 |

Mortality abroad, 45 per cent.

AMPUTATION LOWER 3D OF THIGH ABROAD, INTERMEDIARY AND SECONDARY COMBINED.

| AUTHORITIES. | CASES. | DIED. |
| --- | --- | --- |
| Circular No. 3, Surg. Gen. U. S. A. | 3 | 0 |
| Rept. Boston City Hosp., Dr. Cheever | 4 | 1 |
| " Mass. Gen. Hosp., 1871 | 8 | 6 |
| " U. S. Marine Hosp. | 1 | 0 |
| Dr. Herrgolt, Siege of Strassbugh, 1870–71 | 1 | 1 |
| | 43 | 28 |
| Totals | 60 | 36 |

Mortality abroad, 60 per cent.

AMPUTATION LOWER 3D OF THIGH ABROAD, PATHGLOGICAL.

| AUTHORITIES. | CASES. | DIED. |
|---|---|---|
| Circular No. 3, Surg. Gen. U. S. A. | 2 | 0 |
| Rept. Boston City Hosp., Dr. Cheever | 7 | 2 |
| " Mass. Gen. " 1871 | 101 | 19 |
| " Rostoch " | 3 | 0 |
| " British Army | 2 | 1 |
| * Glasgow Infirmary | 92 | 19 |
| * St. Thomas Hosp., 1835–40 | 13 | 4 |
| * Univ. College Hosp., 1843 | 54 | 10 |
| * Hussey | 55 | 10 |
| * James at Exeter | 119 | 10 |
| * Cases in Med. Times and Gazette, 1851–57 | 54 | 9 |
| * Addenbrooke's Hosp., Cambridge | 92 | 17 |
| * St. George's Hosp., 1866 | 12 | 6 |
| * London Hosp., 1854–57 | 169 | 38 |
| * Provincial Hosps. | 134 | 33 |
| Totals | 909 | 178 |

Mortality Abroad, 20 per cent.

AMPUTATION THIGH ABROAD, TRAUMATIC, PRIMARY, PLACE NOT STATED.

| AUTHORITIES. | CASES. | DIED. |
|---|---|---|
| Dr. E. Warren's surg., p. 395, Confederate Army | 25 | 9 |
| Guy's Hosp. Repts. | 12 | 5 |
| St. Thomas' Hosp. | 5 | 2 |
| St. Bartholomew's Hosp., 1853–71 | 26 | 9 |
| Penn. Hosp. | 24 | 10 |
| Mass. Gen. Hosp. | 60 | 25 |
| Malgaigne, quoted in Gant's Surg., p. 689 | 46 | 34 |
| Other cases " " " " | 24 | 24 |
| Birmingham Gen. Hosp., 1853–64 | 19 | 13 |
| Deutsches Zeitschrift f. Chir. B. 1, S. 187, German-French War, | 23 | 15 |
| Same work, B. V., S. 26 | 5 | 4 |
| Bech's Kriegschir | 10 | 4 |
| Circular No. 6, Surg. Gen. U. S. A. | 423 | 229 |
| New York Hosp. | 16 | 12 |
| Boston City Hosp. | 21 | 15 |
| Siege of Antwerp, Schmidt's Jahrbücher, B. 156 | 12 | 2 |
| " " Paris, 1830–32 " " " | 3 | 0 |
| German-French War " " " | 27 | 17 |
| Crimean War " " " | 1589 | 1424 |
| Italian " " " " | 109 | 85 |
| German " 1866 " " " | 11 | 5 |
| Totals | 2490 | 1943 |

Mortality, 78 per cent.

* Archiv. Klin. Chir. B. VIII. S. 910. These are all amputations for disease of the knee; they must therefore have been, with few exceptions, in the lower 3d, and are consequently classed as such.

AMPUTATION OF THIGH ABROAD, INTERMEDIARY AND SECONDARY COMBINED, TIME NOT STATED.

| AUTHORITIES. | CASES. | DIED. |
|---|---|---|
| Guy's Hosp. Repts. | 11 | 9 |
| St. Thomas' Hosp. Repts. | 2 | 1 |
| St. Bartholomew's Hosp., 1853–71 | 53 | 29 |
| Birmingham Gen. Hosp., 1853–64 | 67 | 15 |
| Gant's Surg., p. 689, Military cases | 300 | 270 |
| Billroth and others, in German-French War | 34 | 22 |
| Bech's Kriegschirurgie | 41 | 22 |
| Circular No. 6, Surg. Gen. U. S. A. | 638 | 477 |
| Mass. Gen. Hosp. | 15 | 9 |
| New York " | 14 | 6 |
| Boston City " | 4 | 3 |
| Pennsylvania " | 15 | 6 |
| Siege of Antwerp, Schmidt's Jahrbücher, B. 156 | 3 | 1 |
| Paris, 1830–32 " " " | 6 | 4 |
| German-French War " " " | 52 | 39 |
| Crimean " " " " | 221 | 197 |
| Italian " " " " | 128 | 107 |
| German War, 1866 " " " | 47 | 28 |
| Dr. E. Warren's Surg., Confederate Army | 39 | 34 |
| Totals | 1690 | 1279 |

Mortality abroad, 76 per cent.

AMPUTATION OF THIGH ABROAD, PATHOLOGICAL, PLACE NOT STATED.

| AUTHORITIES. | CASES. | DIED. |
|---|---|---|
| Guy's Hosp. Repts., 1861–68 | 83 | 27 |
| St. Thomas Hosp. Repts. | 9 | 1 |
| St. Bartholomew's Hosp. Repts., 1853–71 | 278 | 89 |
| St. George Hosp., 1864–68 | 54 | 25 |
| London Hosp., 1862–68 | 68 | 23 |
| King's College Hosp., 1863–68 | 14 | 5 |
| Royal Free " 1862–68 | 6 | 1 |
| Westminster " 1861–67 | 5 | 4 |
| St. Mary's " | 6 | 1 |
| Brit. Army Med. Rep. | 3 | 0 |
| Mr. H. D. Cardin, of Worcester | 6 | 0 |
| New York Hosp. | 21 | 6 |
| Pennsylv. " | 37 | 9 |
| Boston City " | 16 | 4 |
| Mass. Gen. " | 162 | 34 |
| Totals | 768 | 229 |

Mortality abroad, 30 per cent.

The following are figures from various authorities, in which the particulars of time, place, etc., are more imperfectly given than the above:

AMPUTATION OF THIGH, DETAILS, TIME, PLACE AND CAUSE IMPERFECTLY STATED.

| AUTHORITIES. | CASES. | DIED. | PER CENT. MORTALITY. |
|---|---|---|---|
| Military cases from American and various European Wars, after deducting figures previously quoted | 2156 | 1444 | 67 |
| Civil Cases from various Sources | 1243 | 713 | 57 |
| Totals | 3399 | 2157 | 63 |

GENERAL SUMMARY OF AMPUTATIONS OF THE THIGH.

| TOTALS. | LAKE STATES. | | | ABROAD. | | |
|---|---|---|---|---|---|---|
| | CASE | DIED | PER CT. MORT | CASE | DIED | PER CT. MORT |
| Upper 3d, primary | 5 | 2 | | 40 | 24 | 60 |
| " intermediary and secondary | 1 | 1 | | 18 | 8 | 44 |
| " pathological | 2 | 0 | | 30 | 8 | 27 |
| Middle 3d, primary | 4 | 3 | | 33 | 16 | 50 |
| " intermediary and secondary | 5 | 1 | | 39 | 23 | 59 |
| " pathological | 7 | 2 | | 56 | 12 | 22 |
| Lower 3d, primary | 18 | 5 | 28 | 83 | 37 | 45 |
| " intermediary and secondary | 9 | 3 | 33 | 60 | 36 | 60 |
| " pathological | 22 | 0 | 00 | 909 | 178 | 20 |
| Place not stated, primary | | | | 2490 | 1943 | 78 |
| " " " intermediary and secondary | | | | 1690 | 1279 | 76 |
| " " " pathological | | | | 768 | 229 | 30 |
| Conditions not stated at all | 3 | 1 | | 3399 | 2157 | 63 |
| Totals | 76 | 18 | 24 | 9615 | 5950 | 62 |

It appears, therefore, that the average mortality of amputation of the thigh, in the Lake States, is considerably less than half that given in the published statistics elsewhere.

OPINIONS OF AUTHORS.

Authors contradict each other somewhat as to the conditions requiring amputation of the thigh.

Erichsen, vol. II., pp. 200 and 237, advises immediate amputation of all compound gun shot fractures of the femur, except in the upper third.

On the other hand the *Archives Générales de Médicene* (tome xiii., serie 5e.) says that in the Crimean War conservative treatment of gun shot fractures of the femur, or of any other part of the inferior member, was five times more successful than amputation. Yet Macleod, discussing the same war, says, we ought to use conservative treatment in the upper third, and amputation in the middle and lower thirds.

Hamilton says, in gun shot fractures of the middle third conservative treatment and amputation have equal success, while conservative treatment is the most fatal in the lower third. This is doubtless because gun shot fractures in the lower third are apt to split into the knee joint, thus opposing a very dangerous complication to conservative success.

In contradiction to this difference of the upper and lower thirds, Max Schmidt (*Schmidt's Jahrbücher*, 1872) says all the war statistics of 1830 show that conservative treatment of gun shot fractures of the thigh is more successful than amputation, *in every portion* of the member.

Demme and Legouest give statistics to the same end (see same article), showing that in all parts of the thigh, treated for gun shot fractures, the mortality of amputation exceeded that of conservative treatment by the following amounts:

| | | | | | DEMME. | LEGOUEST. |
|---|---|---|---|---|---|---|
| Mort. of amp. | in | upper 3d | exceeds | conser. treat. by | 29 pr. ct. | 27 pr. ct. |
| " | " | middle 3d | " | " | 11 " | 26 " |
| " | " | lower 3d | " | " | 18 " | 32 " |

Legouest elsewhere states (*Chirurgie d'Armée*, p. 537), that in the battle of Langensalza, 1866, and in the French army in the Crimea, and in Italy, conservative treatment of the thigh was most successful by about nineteen per cent.; while in the English army in the Crimea, in the American war of secession, in the Schleswig-Holstein war and in Stromeyer's figures from the battle of Langensalza, amputations of the thigh were more successful than conservative treatment by about fourteen per cent.

Dr. Albert Malinas (Conservation, Paris, 1872, p. 51) gives a table showing that gun shot fractures in the thigh, in the Crimea and in the Italian war, according to the experience of the French army, were better treated conservatively than by amputation. He says the results were these:

| | MORTALITY OF CONSERVATIVE TREATMENT OF THIGH. | MORTALITY OF AMPUTATION OF THIGH. |
|---|---|---|
| Crimean War | 35 per cent. | Upper 3d, 94 per cent.<br>Middle 3d, 94 per cent.<br>Lower 3d, 90 per cent. |
| Italian War | 58 per cent. | 64 per cent. |

Dr. S. W. Gross, in the October number of the *Am. Jour. Med. Sci.*, 1867, carefully collated the statistics on this subject, from which essay I condense the following points respecting gun shot fractures of the thigh, treated some by amputation and some by conservative treatment:

TREATMENT OF GUN SHOT FRACTURES OF THE THIGH.

AMPUTATIONS.

| | | CASES. | DIED. | PER CENT. MORTALITY. |
|---|---|---|---|---|
| All kinds Combined | | 4123 | 3146 | 76 |
| Primary | | 695 | 381 | 55 |
| Secondary (and Intermediary) | | 753 | 572 | 76 |
| Franco-Sardinian Army in Italy, and British Army in Crimea. | Upper 3d. | 225 | 177 | 79 |
| | Middle 3d. | 268 | 175 | 65 |
| | Lower 3d. | 236 | 130 | 55 |

CONSERVATIVE TREATMENT.

| | | CASES. | DIED. | PER CENT. MORTALITY. |
|---|---|---|---|---|
| Franco-Sardinian Army in Italy, in 1859, French Army in Crimea and Am. War of Secession. | All kinds combined | 1450 | 923 | 64 |
| | Upper 3d | 445 | 306 | 69 |
| | Middle 3d | 327 | 181 | 55 |
| | Lower 3d | 295 | 150 | 51 |

He concludes that in gun shot fractures of the thigh, conservative treatment is better than amputation by twelve per cent., the lower third being no exception, and better than exsection of the femur by twenty-four per cent.

Billroth, of Vienna, in his letters from the late German-French war, collates figures from various wars, which foot up as follows:

| CONSERVATIVE TREATMENT OF GUN SHOT FRACTURE OF THIGH. | | MILITARY AMPUTATION OF THIGH. | |
|---|---|---|---|
| Cases | 1339 | Cases | 3721 |
| Died | 949 | Died | 2826 |
| 71 per cent. mortality. | | 76 per cent. mortality. | |

As already stated, the *Archives Générales de Méd.*, 1859, has an article claiming that, in the Crimean war, conservative treatment for gun shot fracutre of the leg and thigh was five times more successful than amputation. It is evident that the opinions of the most eminent men are in utter contradiction on this subject; and by some inexcusable blundering the figures are in the same situation. The truth is that military statistics are often extremely delusive, in consequence of the improper manner in which they are collected. We will discuss this matter further under the head of "Conclusions."

Formerly all gun shot fractures of the femur were supposed to demand amputation, but Malgaigne defended, before the French Academy, the opinion that conservative treatment should be tried wherever the circumstances did not compel amputation. Velpeau and Jobert (de Lamballe) sustained him.

Hamilton (*Military Surgery*, p. 399) advises to amputate the thigh for gun shot fracture: 1. When the patient must be carried far, over rough roads without adequate support to limb. 2. When the bones are greatly comminuted. 3. When there are uncontrollable pains and spasms. 4. When there is great contusion or laceration of soft parts. 5. When the principal arteries or nerves are destroyed. 6. When the fracture is at or near the knee.

He advises not to amputate: 1. When the bullet fractures the head, neck—trochanter—or shaft just below the trochanter. 2. When the wound is from a pistol, a spent ball, or any projectile which makes but little comminution.

Longmore, in his article in *Holmes' System of Surgery*, vol. ii, p. 227, quotes the statistics of the American war as showing that conservative treatment of gun shot fractures of the upper third of the thigh was three per cent. more successful than amputation, and hence recommends conservatism in uncomplicated cases. In the middle and lower thirds he recommends amputation, as shown by statistics to be slightly safer in the middle, and decidedly safer in the lower third than conservative treatment. His figures from Circular No. 6, S. G. O., are so erroneously quoted that I am obliged to correct them from the original document:

| | CONSERV. TREAT. | | | AMPUTATIONS. | | |
|---|---|---|---|---|---|---|
| | CASE | DIED | PER CT. MORT | CASE | DIED | PER CT. MORT |
| Upper third | 330 | 237 | 72 | 32 | 24 | 75 |
| Middle third | 238 | 132 | 55 | 93 | 51 | 55 |
| Lower third | 173 | 101 | 58 | 243 | 112 | 46 |

Circular No. 6, of the U. S. Surg. Gen., compares conservative treatment of gun shot fractures of the knee, with treatment by amputation just above, with the following results:

| | CASES. | DIED. | PER CENT. MORTALITY. |
|---|---|---|---|
| Gun Shot Fracture of Knee, treated by Amp. Lower Third of Thigh | 452 | 331 | 73 |
| Same Injury Conservatively Treated | 308 | 258 | 84 |

Dr. E. Warren, of the Confederate army, and Surg. Gen. of North Carolina, gives, in his "Surgery of the Field and Hospital," two hundred and one cases of fractured knee joint from the Richmond hospitals, with one hundred and twenty-one deaths, a mortality of sixty per cent. He remarks judiciously that these figures do not represent the whole truth, as many bad cases died before reaching the hospitals. Were these added the mortality would doubtless be greater.

The *Deutsch Zeitschrift für Chir.*, Bd. 2, S. 106, gives, from the German-French war, thirty-four cases of penetrating gunshot wounds of the knee joint, with twenty-four deaths; a mortality of seventy per cent.

Max Schmidt (*Jahrbücher*, 1872) advocates conservative treatment—not only in wounds about the knee, not penetrating the capsule, but also in the simpler intracapsular wounds.

Surgeon J. M. Woodworth, formerly Med. Director of the Army of the Tennessee, and now Supervising Surgeon of Marine Hosps., claims, on the contrary, that almost all gun shot openings of the knee joint, even if the bones are not fractured, should, in military practice, be amputated.

Guthrie (Commentaries on the Crim. War) says gun shot fractures of the knee joint imperatively require primary amputation; but that if the patella alone be broken, and that only moderately, delay may be allowed. At page 151 he maintains that when gun shot fractures of the lower half of the femur do not communicate with the knee joint, conservative treatment should always be preferred.

### CONCLUSIONS.

From this somewhat contradictory mass of opinions on one of the plainest operations in surgery we see how far from being settled many precepts of our art still are. We will try to evolve partial order out of the chaos, and where this is impossible we will at least ascertain what points are still unknown, and must wait the further growth of science for light upon them.

1. It is settled forever, as every one knows, that the nearer the operation comes to the body the greater the risk, other things being equal. There is an apparent exception in the traumatic secondary cases, for in these the mortality of secondary cases increases as we approach the knee. This is probably due to the inclusion of many cases in which compound fractures opened that joint, producing suppuration, etc., in which accident the earlier secondary amputations are excessively fatal. Amputations in the height of an active suppurating inflammation of the knee are considered almost necessarily fatal. Did the published records admit of our sifting out these knee cases, we should probably find that the remaining secondary cases followed the usual rule of increasing danger as we approach the body.

Our average Lake State mortality for all amputations of the thigh is only 24 per cent. against 62 per cent. elsewhere. Our number in the upper two-thirds are too small to establish reliable averages, but if we distribute the 24 per cent. risk according to the experiences elsewhere, we shall have the following as our probable rates:

PROBABLE RISK OF AMPUTATIONS OF THIGH IN THE LAKE STATES.

| | | | | | | |
|---|---|---|---|---|---|---|
| Upper | 3d | Primary | about | 30 | per | cent. |
| " | 3d | Intermediary | " | 45 | " | " |
| " | 3d | Purely secondary | " | 20 | " | " |
| " | 3d | Pathological | " | 18 | " | " |
| Middle | 3d | Primary | " | 24 | " | " |
| " | 3d | Intermediary | " | 36 | " | " |
| " | 3d | Purely secondary | " | 25 | " | " |
| " | 3d | Pathological | " | 15 | " | " |
| Lower | 3d | Primary | " | 22 | " | " |
| " | 3d | Intermediary | " | 45 | " | " |
| " | 3d | Purely secondary | " | 25 | " | " |
| " | 3d | Pathological | " | 10 | " | " |

These figures can only be approximate, of course. Massive as are the published statistics of amputations of the thigh abroad, most of them are in such a wretchedly crude and even contradictory condition, that their usefulness is in a great measure lost, and proportions taken from them and applied to our cases must be received with many allowances.

I ought to say here that in the division into traumatic and pathological, experience shows that amputations of "expediency," or "complaisance," that is, amputations performed to remove deformities, on limbs otherwise healthy, have a mortality much greater than other pathological cases, and rank nearly the same as traumatic primary operations.

The above averages will do as a starting point, but in each case we must consider the individual modifying circumstances. If the patient's condition and surroundings are better than usual, his risk will be much less; and if the reverse, it will, of course, be greater than the above average.

Injuries which, like bullet wounds, comminute the bones in the interior of the knee, require primary amputation, but if the period for this has already passed, the patient is in a very dangerous situation, as amputations of these cases are desperately perilous during the acute portion of the suppurative stage, and excisions are the same, while delay is not much better. Perhaps the best way would be to open the joint, pick out the fragments, apply Lister's carbolic acid treatment thoroughly every day , and keep up extension of the leg by adhesive plasters, weight and pulley, and thus carry the case over the period of acute activity, when the risk of an amputation will be greatly diminished.

This is only a suggestion, for which there is no accumulation of experimental proof as yet adduced.

Ordinary compound fractures, not comminuted, but yet extending into the knee joint, were often best treated in former times by a primary amputation; but at present, he that is master of the antiseptic methods, and bold enough to apply them thoroughly, will find them more useful than amputation for such cases, if seen immediately.

Military fractures of the thigh, not implicating the knee joint, and not accompanied with such injuries to vessels or other parts as will produce mortification of the member, are best treated conservatively, and especially so in the upper half of the thigh.

(To be Continued.)

---

The following resolutions were passed at the Annual Meeting, April 11, 1876, of the Michigan State Board of Health. The Journal and Examiner endorses them with the most hearty approval:

Whereas, The Signal Service Bureau of the United States has demonstrated its great usefulness in securing benefits to public safety of life in this State, particularly to the large number of persons employed upon or journeying over the great lakes, and in promoting health through better protection of cereal and other food crops because of its warnings, and also through the valuable data for the study of the relations of health and of diseases to the climatic conditions, knowedge of which is essential to an avoidance of causes now statistically shown to be of great influence on the death rate; therefore,

*Resolved*, That the hope be expressed by this Board that the means of the usefulness of U. S. Signal Service Bureau be in no way abridged, but rather increased; that it be permanently organized, and that its sphere of labor be enlarged, especially in the direction of obtaining and recording meteorological data bearing still more closely upon important questions relating to the public health.

*Resolved*, That, although not essential in connection with its work for the prediction of storms, it is desirable for purposes of progress in public health that we have at least monthly statements of the absolute humidity of the atmosphere, and of the exact atmospheric pressure at different stations (not calculated to sea level as required for other purposes) and that it is also desirable that observations on Ozone be recorded.

*Resolved*, That in the opinion of this Board, such an enlargement of the means and labor of the Signal Service as is contemplated in the foregoing, will add to its present acknowledged usefulness, and is desirable in the interests of public health in this State.

*Resolved*, That the Secretary of this Board be directed to forward a copy of the foregoing preamble and resolutions to the Chief Signal Service Officer of the United States, and to each of the members of Congress from this State.

